# Complete Nucleotide Sequence of pGA45, a 140,698-bp IncFII_Y_ Plasmid Encoding *bla*_IMI-3_-Mediated Carbapenem Resistance, from River Sediment

**DOI:** 10.3389/fmicb.2016.00188

**Published:** 2016-02-24

**Authors:** Bingjun Dang, Daqing Mao, Yi Luo

**Affiliations:** ^1^School of Environmental Science and Engineering, Tianjin UniversityTianjin, China; ^2^Ministry of Education Key Laboratory of Pollution Processes and Environmental Criteria, College of Environmental Science and Engineering, Nankai UniversityTianjin, China

**Keywords:** carbapenem resistance, plasmid, pGA45, T6SS, antibiotic resistance

## Abstract

Plasmid pGA45 was isolated from the sediments of Haihe River using *Escherichia coli* CV601 (*gfp*-tagged) as recipients and indigenous bacteria from sediment as donors. This plasmid confers reduced susceptibility to imipenem which belongs to carbapenem group. Plasmid pGA45 was fully sequenced on an Illumina HiSeq 2000 sequencing system. The complete sequence of plasmid pGA45 was 140,698 bp in length with an average G + C content of 52.03%. Sequence analysis shows that pGA45 belongs to IncFII_Y_ group and harbors a backbone region which shares high homology and gene synteny to several other IncF plasmids including pNDM1_EC14653, pYDC644, pNDM-Ec1GN574, pRJF866, pKOX_NDM1, and pP10164-NDM. In addition to the backbone region, plasmid pGA45 harbors two notable features including one *bla*_IMI-3_-containing region and one type VI secretion system region. The *bla*_IMI-3_-containing region is responsible for bacteria carbapenem resistance and the type VI secretion system region is probably involved in bacteria virulence, respectively. Plasmid pGA45 represents the first complete nucleotide sequence of the *bla*_IMI_-harboring plasmid from environment sample and the sequencing of this plasmid provided insight into the architecture used for the dissemination of *bla*_IMI_ carbapenemase genes.

## Introduction

The overuse and misuse of antibiotics have contributed to the emergence and spread of antibiotic resistance genes (ARGs) and multidrug resistance pathogens (Zhang and Zhang, 2011; He et al., 2014). Now ARGs have been recognized as a new type of pollutants (Pruden et al., 2006). Among various ARGs, carbapenem resistance genes, especially plasmid mediated carbapenem resistance genes, have raised worldwide concern, leading to the extensive research on some of these genes and related plasmid architecture(McGann et al., 2012; Tiwari et al., 2012; Villa et al., 2012, 2013; Lo et al., 2013; Tiwari and Moganty, 2014). Acquired carbapenem resistance can be resulted from carbapenemases of Amber class A (IMI, GES and KPC), Amber class B (metallo β-lactamases including IMP, VIM and NDM) or Amber class D (OXA-48 and OXA-181) (Nordmann et al., 2012). The *bla_KPC_* gene of Amber class A and the metallo β-lactamase genes have been the research focus but there is rare reports about the other carbapenem resistance genes especially little is known about the *bla*_IMI_ genes and the plasmid architecture involved in the dissemination of this type of genes (Aubron et al., 2005; Yu et al., 2006; Rojo-Bezares et al., 2012; Teo et al., 2013; Chen et al., 2015).

The *bla*_IMI-1_ gene was first identified in 1996 and found to be located in the chromosome of *Enterobacter cloacae* isolates whereas *bla*_IMI-2_ was first identified in 2005 in *Enterobacter asburiae* isolates and found to be related to plasmids (Rasmussen et al., 1996; Aubron et al., 2005). In 2009, a new variant of *bla*_IMI-1_, *bla*_IMI-3_, was identified in Hong Kong in *Enterobacter cloacae* isolates (Chu et al., 2011). The *bla*_IMI-3_ was also located in the conjugative plasmids. The *bla*_IMI_-mediated carbapenem resistance is an infrequent mechanism but it has been reported both in clinical strains and environmental bacteria from rivers. Horizontal transfer may occur between environmental bacteria and clinical strains. With the horizontal transfer, the *bla*_IMI_ genes would broaden their hosts and inevitably pose serious risks to the public health. For further research on the dissemination of *bla*_IMI_ genes, the full sequence of *bla*_IMI_-related plasmid is needed. Here we report the first complete nucleotide sequence of *bla*_IMI_-carrying conjugative plasmid from the environment sample.

## Materials and Methods

### Studying Sites and Sample Collection

Sediment sample was collected under JinGang Bridge of Haihe River. JinGang Bridge was located in densely populated urban areas with frequent human activities. Sediment sample was collected with a grab sampler and then put into sterile containers. The sample was immediately taken to the laboratory and stored in -20°C for subsequent experiments after sampling was completed.

### Isolation of the Conjugative Plasmids Conferring Resistance to Imipenem

In order to obtain conjugative plasmids which confer resistance to imipenem, amipicillin resistant plasmids were first isolated by filter mating assays and these ampicillin resistant plasmids were then subjected to antibiotic susceptibility testing against imipenem and other antibiotics. The filter mating assays were applied using *Escherichia coli* CV601 (*gfp*-tagged, kanamycin and rifampicin resistant) as recipients and sediment samples as donors (Heuer et al., 2002). Transconjugants were selected by Mueller-Hinton agar plates supplemented with ampicillin (100 mg L^-1^), kanamycin (50 mg L^-1^), rifampicin (50 mg L^-1^) and cycloheximide (100 mg L^-1^). *E. coli* CV601 recipient culture was plated on the same selective plates as controls. The procedure used for filter mating assays was described by Heuer et al. (2002) with slight modification. Briefly, the sediment samples from which the indigenous bacteria were used as donors were doubled to 2 g and the Luria-Bertani (LB) broth used for resuspending the sediment samples was accordingly doubled to 18 ml. After incubation for 2 days, transconjugants were determined by green fluorescence which is resulted from green fluorescence protein (GFP) gene. All the ampicillin resistant transconjugants were then streaked on the ampicillin selective plates. Overnight culture of these transconjugants were stored in -80°C for further study.

### Antibiotic Susceptibility Testing of the Ampicillin Resistant Transconjugants

Kirby-Bauer disk diffusion method was applied to determine which ampicillin resistant transconjugants confer resistance to imipenem. According to the criteria of the Clinical and Laboratory Standards Institute (CLSI), the disks used in this study are as follows: imipenem (10 μg), ampicillin (10 μg), gentamicin (10 μg), streptomycin (10 μg), tetracycline (30 μg), ciprofloxacin (5 μg), sulfamethoxazole (300 μg) and erythromycin (15 μg). *E. coli* ATCC25922 was used as quality control strain. In this study, one transconjugant designated GA45 was found to confer resistance to imipenem and ampicillin. The conjugative plasmid harbored by GA45 was named pGA45 and stored for further analysis.

### Conjugative Transfer Experiments and the Role Determination of pGA45 in Recipient Strains

To assess the conjugative frequency of plasmid pGA45, liquid mating assays were employed using *E. coli* CV601 (pGA45) as donor strains and *E. coli* J53 (azide and nalidixic acid resistance) as recipient strains. For liquid mating assay, overnight cultures of donor and recipient strains were centrifuged, washed and adjusted to the optical density of 0.6 at the wavelength of 600 nm (OD_600_) with LB broth. Then 0.5 ml cultures of each donor and recipient strains were mixed and make up to the volume of 5 ml with LB broth. After incubation of 16 h in 37°C, transconjugants were selected on LB plates containing azide (200 mg L^-1^), nalidixic acid (20 mg L^-1^) and ampicillin (100 mg L^-1^). Conjugative frequency was determined by the following formula: conjugative frequency = transconjugants (CFU/ml)/recipients (CFU/ml). The *E. coli* J53 transconjugants were then tested against imipenem to confirm the role of pGA45 in recipient strains. The results showed that pGA45 also conferred resistance to imipenem in recipient strains.

### Plasmid Sequencing and Bioinformatics

Plasmid DNA from the *E. coli* J53 transconjugants was extracted using a Qiagen plasmid midikit (Qiagen, Inc). The plasmid DNA was sequenced on an Illumina HiSeq 2000 sequencing system. Sequencing reads were *de novo* assembled into contigs using the SOAPdenovo 2.04 software (Li et al., 2008, 2010). Gaps between contigs were closed by PCR with standard Sanger sequencing. Glimmer 3.02 was used to predict putative open reading frames (ORFs) (Salzberg et al., 1998; Delcher et al., 1999, 2007). All ORFs were translated and aligned with different protein databases including NR (version: 20121005), KEGG (version: 59), COG (version: 20090331), SwissProt (version: 201206) and GO (version: 1.419).

### Nucleotide Sequence Accession Number

The complete nucleotide sequence of pGA45 was deposited in GenBank under accession no. KT780723.

## Results

**FIGURE 1:**
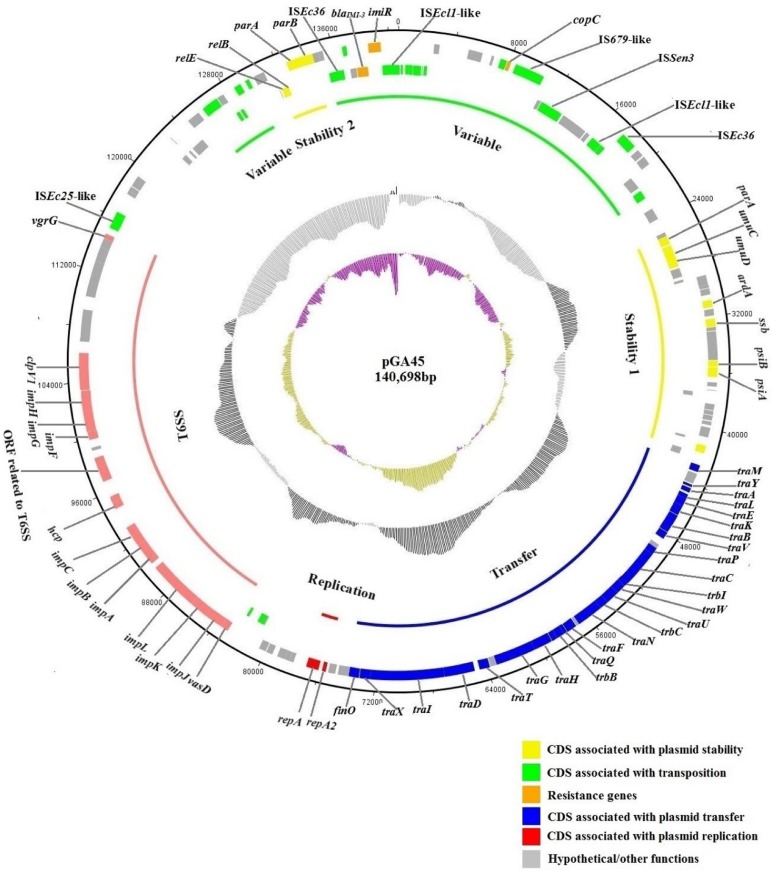
**Circular map of plasmid pGA45 (GenBank KT780723).** The rings show from outside to inside (i) position of predicted coding sequences in the clockwise direction, (ii) position of predicted coding sequences in the counterclockwise direction, (iii) different regions of plasmid pGA45, (iv) GC plot in a 10,000-bp window, (v) GC skew in a 10,000-bp window. Each predicted coding sequence is color-coded by its function as shown in the figure.

Sequencing of plasmid pGA45 generated 237,937,000 reads in total. Reads either of low quality or representing the host chromosome contamination through comparison with the sequence of reference strain *E. coli* MG1655 were filtered. In the end, 34 contigs were obtained and then assembled into 10 scaffolds. Through PCR and Sanger sequencing, gaps between contigs and scaffolds were closed. The complete sequence of plasmid pGA45 was 140,698 bp in length with an average G + C content of 52.03% (**Figure 1**). Analysis of the sequence identified 157 ORFs, 64 of which were transcribed in the opposite direction. The backbone of this plasmid included the replication region, stability region 1, and transfer region (51524 bp), making up 36.6% of the total sequence. This backbone region shared high homology and gene synteny to several other IncF plasmids including pNDM1_EC14653 (Wu et al., 2015), pYDC644 (GenBank accession no.: KR351290), pNDM-Ec1GN574 (GenBank accession no.: KJ812998), pRJF866 (Qu et al., 2015), pKOX_NDM1 (Huang et al., 2013) (86% query coverage and 97% nucleotide identity, NCBI database) and pP10164-NDM (Sun et al., 2015) (86% query coverage and 94% nucleotide identity, NCBI database). Notably, all these plasmids were recently sequenced and four of them were found in China. Thus, to our best of knowledge, pGA45 and other recently sequenced plasmids represent a new IncF subtype and this type of plasmids exhibit a high prevalence in China. By contrast, the rest parts of pGA45 [including the variable region, the stability region 2 and the type VI secretion system (T6SS) region] showed no significant similarities with other sequenced plasmids in GenBank.

The replication region (1,431 bp) of pGA45 (positions 75496–76926), including the replication initiation protein gene *repA* and replication regulatory protein gene *repA2*, shared 93% nucleotide similarity with the six IncF plasmids mentioned above with 100% query coverage. Plasmid pGA45 was further assigned to the IncFII_Y_ incompatibility group through sequence queries against the plasmid MLST databases^1^.

Plasmid pGA45 contained one transfer region (position 42643–73867 bp) that comprised 21 *tra* genes, 3 *trb* genes (ordered as follows: *traM*, *traY*, *traA*, *traL*, *traE*, *traK*, *traB*, *traV*, *traP*, *traC*, *trbI*, *traW*, *traU*, *trbC*, *traN*, *traF*, *traQ*, *trbB*, *traH*, *traG*, *traT*, *traD*, *traI*, *traX*) and *finO*. Mating out experiments demonstrated that this region made pGA45 self-transmissible at a relatively high frequency of (7.81 ± 7.15) × 10^-3^ transconjugants per recipient between *E. coli* CV601 and *E. coli* J53.

The genes on plasmid pGA45 that are responsible for plasmid stability and maintenance included *umuC-umuD* genes which confer resistance to UV light, *relE-relB* genes encoding a toxin-antitoxin system, *ardA* gene with antirestriction function, *parA-parB* genes for partition, *psiA-psiB* genes involved in the bacterial SOS inhibition and *ssb* gene involved in recombination and repair.

The variable region of plasmid pGA45 contained two resistance genes including one ARG *bla*_IMI-3_ and one copper resistance gene *copC* (with 54% coverage and 94% amino acid identity to *Klebsiella pneumoniae* subsp. pneumoniae DSM 30104, NCBI database). Plasmid pGA45 also harbored a T6SS region (position 82987–114739bp) which may be related to bacterial pathogenesis.

## Discussion

**FIGURE 2:**
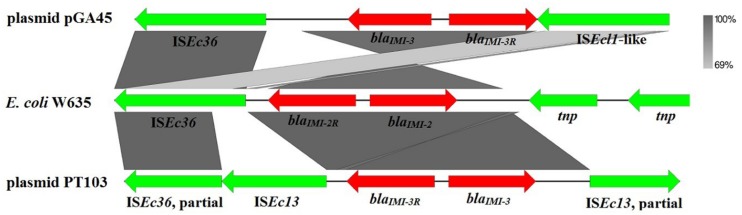
**Schematic representation and comparison of *bla*_IMI_-containing genetic structures.** Gray shading represents shared regions among three *bla*_IMI_-containing structures. Genes and mobile elements are color-coded based on their functions. Mobile elements are displayed using green arrows while antibiotic resistance genes are shown by red arrows. GenBank accession no.: plasmid pGA45, KT780723; *E. coli* W635, JN412066.1; plasmid pT103, NG_036022.1.

The *bla*_IMI-3_ gene from the variable region was the only ARG harbored by pGA45. The *bla*_IMI-3_-containing region (position 58145–74139 bp) was bracketed by one copy of insertion sequence IS*Ec36*^2^ and one copy of insertion sequence highly similar to IS*Ecl1*^2^ (with 100% coverage and 94% nucleotide identity to IS*Ecl1*) in the same orientation (**Figure 2**). IS*Ec36* was first identified in a *bla*_IMI-2_-bearing *E. coli* W635 strain (Rojo-Bezares et al., 2012). In this strain, *bla*_IMI-2_ was detected upstream the IMI-2R gene. However, in plasmid pGA45, *bla*_IMI-3_ and IMI-3R changed positions with each other and IMI-3R was located upstream the *bla*_IMI-3_ gene. Another well characterized *bla*_IMI_-containing structure was from *Enterobacter cloacae* plasmid pT103 (GenBank accession no.: NG_036022.1) (Yu et al., 2006). In this partially sequenced plasmid, the *bla*_IMI_-containing region comprises two IS*Ec13*^2^ (one is partial) elements flanking the *bla*_IMI-3_ and *bla_IMI-3R_* genes in the opposite directions and one partial IS*Ec36* located downstream of this region. In all these characterized *bla*_IMI_-containing regions, *bla*_IMI_ genes have close relationships with insertion sequence IS*Ec36*. Therefore, IS*Ec36* may play an essential role in the dissemination of *bla*_IMI_ genes between different plasmids. It is also noteworthy to point out that the six plasmids mentioned above similar to pGA45 in backbones mainly have two different bacterial hosts, *Klebsiella pneumoniae* and *Enterobacter cloacae.* In addition, the identified *bla*_IMI_ genes were mostly from *Enterobacter* species. In view of this, the most probable hosts for plasmid pGA45 were *Enterobacter* species.

Another notable feature harbored by pGA45 was the T6SS region. Compared to other similar IncFII plasmids, the T6SS region was unique to pGA45. This region was most closely related to the T6SS system of plant pathogen *Erwinia amylovora* not only in nucleotide identity (71% coverage and 82% nucleotide identity) but also in gene organization. Previous studies showed that *bla*_IMI-2_ and *bla*_IMI-3_ genes were located on plasmids with sizes ranging from 48.5 to 80 kb (one of these plasmids had been identified to belong to IncF group). In this study, pGA45 was much bigger than these plasmids. This perhaps resulted from the integration of this T6SS region (31753 bp). The T6SS region of plasmid pGA45 was flanked by a copy of IS*Ec25*-like element (with 100% coverage and 85% nucleotide identity to IS*Ec25*) downstream and two transposase genes (with weak amino acid identity to known transposase) upstream. The T6SS region of plasmid pGA45 comprised 14 T6SS-related genes including *vasD* (with 93% coverage and 64.46% amino acid identity to *Erwinia amylovora* ATCC 49946, KEGG database), *impJ* (with 100% coverage and 82.55% amino acid identity to *Erwinia pyrifoliae* Ep1/96, KEGG database), *impK* (with 99% coverage and 84.26% amino acid identity to *Erwinia pyrifoliae* Ep1/96, KEGG database), *impL* (with 100% coverage and 84.38% amino acid identity to *Erwinia tasmaniensis*, KEGG database), *impA* (with 99% coverage and 77.45% amino acid identity to *Erwinia amylovora* ATCC 49946, KEGG database), *impB* (with 97% coverage and 86.52% amino acid identity to *Erwinia amylovora* ATCC 49946, KEGG database), *impC* (with 100% coverage and 94.99% amino acid identity to *Erwinia billingiae*, KEGG database), *hcp* (with 99% coverage and 86.79% amino acid identity to *Enterobacter cloacae subsp. cloacae* ATCC 13047, KEGG database), an ORF encoding a FHA-domain containing protein (with 97% coverage and 70.38% amino acid identity to *Erwinia pyrifoliae* Ep1/96, KEGG database), *impF* (with 99% coverage and 85.03% amino acid identity to *Erwinia amylovora* ATCC 49946, KEGG database), *impG* (with 100% coverage and 87.5% amino acid identity to *Erwinia pyrifoliae* Ep1/96, KEGG database), *impH* (with 99% coverage and 76.95% amino acid identity to *Erwinia tasmaniensis*, KEGG database), *clpV* (with 99% coverage and 89.64% amino acid identity to *Erwinia amylovora* ATCC 49946, KEGG database) and *vgrG* (with 100% coverage and 96.92% amino acid identity to *Kosakonia radicincitans* DSM 16656, NR database).

The T6SS was a recently discovered phage-like secretion apparatus. First reported in *Vibrio cholerae* and *Pseudomonas aeruginosa*, T6SS was likely to be involved in bacterial pathogenesis through acting like a potential nano-syringe for the translocation of effector proteins into the host cell (Pukatzki et al., 2006; Sarris et al., 2011). The Hcp (haemolysin co-regulated protein) and VgrG (valine-glycine repeat G protein) proteins are putative effectors for T6SS (Russell et al., 2014) and the genes encoding these two proteins are also found to be located in the T6SS region of plasmid pGA45. T6SS-related genes are harbored by many kinds of Gram-negative bacterial pathogens which can result in human or animal diseases. In this study, plasmid pGA45 was isolated from river sediment which was collected from urban section of Haihe River. This area was densely populated and strongly affected by human activities. In previous published literatures, most of the *bla*_IMI_ isolates were from in clinical settings. Therefore, the occurrence of T6SS and *bla*_IMI-3_-containing plasmid pGA45 in the river environment are a potential risk for human health and the horizontal transfer of this plasmid between *Enterobacteriaceae* bacteria may aggravate this situation.

## Conclusion

This report demonstrated the complete nucleotide sequence of the *bla*_IMI_-harboring plasmid. The sequencing of this plasmid provided insight into the architecture used for the dissemination of *bla*_IMI_ carbapenemase genes. In addition to the *bla*_IMI_ gene, plasmid pGA45 also harbored a T6SS cluster probably involved in bacteria virulence. Notably, this plasmid was isolated from environment sample, which will increase the risks of obtaining infections resulted from various types of pathogens carrying this plasmid.

## Author Contributions

DM and YL designed experiments; BD carried out experiments and analyzed experimental results; BD wrote the manuscript.

## Conflict of Interest Statement

The authors declare that the research was conducted in the absence of any commercial or financial relationships that could be construed as a potential conflict of interest.

## Footnotes

1 http://pubmlst.org/plasmid

2 https://www-is.biotoul.fr//
